# Differentiating the COVID-19 Infection and Vaccine Experiences of Patients With Systemic, Single Organ, and Overlap Immune-Mediated Inflammatory Disease: Protocol for a Secondary Analysis for Enhancing COVID-19 Vaccine Pharmacovigilance

**DOI:** 10.2196/68785

**Published:** 2026-02-27

**Authors:** Meredith Leston, José M Ordóñez-Mena, Xinchun Gu, Mark Joy, F D Richard Hobbs, Lennard Y W Lee, Ioannis Parodis, Laura Andreoli, Nelly Ziade, Latika Gupta, Vikas Agarwal, Richard Conway, John Isaacs, Jeffrey Curtis, Alessia Alunno, Stefan Siebert, Ori Elkayam, Karen Schreiber, Simon de Lusignan

**Affiliations:** 1 Nuffield Department of Primary Care Health Sciences University of Oxford Oxford United Kingdom; 2 Oxford Institute of Digital Health University of Oxford Oxford United Kingdom; 3 Karolinska University Hospital Stockholm Sweden; 4 University of Brescia Brescia Italy; 5 St Joseph University Beirut Lebanon; 6 The Royal Wolverhampton NHS Trust Wolverhampton United Kingdom; 7 Sanjay Gandhi Post Graduate Institute of Medical Sciences Lucknow India; 8 St James' Hospital Dublin Ireland; 9 Translational and Clinical Research Institute Faculty of Medical Sciences Newcastle University Newcastle upon Tyne United Kingdom; 10 Newcastle NIHR Biomedical Research Centre Newcastle upon Tyne Hospitals NHS Foundation Trust Newcastle upon Tyne United Kingdom; 11 University of Alabama at Birmingham Birmingham, AL United States; 12 Internal Medicine and Nephrology Division MeSVA Department University of L'Aquila L'Aquila Italy; 13 University of Glasgow Glasgow United Kingdom; 14 Department of Rheumatology Tel Aviv Medical Center, Gray Faculty of Medical and Health Sciences Tel Aviv University Tel Aviv Israel; 15 Danish Center for Expertise in Rheumatology (CeViG) Danish Hospital for Rheumatic Diseases Sønderborg Denmark; 16 Institute for Regional Health Southern Danish University Odense Denmark; 17 Thrombosis and Haemostasis Guy’s and St Thomas NHS Foundation Trust London United Kingdom; 18 University of Oxford Oxford United Kingdom

**Keywords:** vaccines, vaccination, pharmacovigilance, surveillance, patient-centered research, adverse events of interest, side effects, digital health, COVID-19

## Abstract

**Background:**

Patients with immune-mediated inflammatory disease (IMID), including autoimmunity, fared substantially worse than the general population during the COVID-19 pandemic, both in terms of infection outcomes and disruption to daily life. Despite this, COVID-19 vaccine uptake has not been universal in this population. The absence of patients with IMID from clinical trials and the subsequent lack of precision in vaccine safety profiling have resulted in vaccine hesitancy in this high-risk group.

**Objective:**

This protocol sets out an investigation that aims to address this by enhancing COVID-19 vaccine pharmacovigilance for patients with IMID. Combining the international data and knowledge assets of the COVID-19 Vaccination in Autoimmune Diseases (COVAD) 1 study and the electronic Delphi Study to Define and Risk-Stratify Immunosuppression (DESTINIES), the objective is to differentiate patient-reported COVID-19 infection and vaccine outcomes between participants with systemic, single organ, and overlap IMID and general population controls.

**Methods:**

The COVAD-1 study successfully collected anonymized data on the demographic, health, COVID-19 infection, and COVID-19 vaccination outcomes of a broad range of participants with IMID between March and December 2021. This protocol expands on this initial analysis by using IMID specialists within the DESTINIES Consortium to allocate survey respondents into single organ and systemic categories and thereby produce comparative vaccine benefit-risk profiles between these and general population controls. Because of the respondents’ ability to self-report multiple diagnoses, an overlap group was introduced for those affected by both single organ and systemic disease. Descriptive statistics and both single and multivariable logistic regressions will be used to test for significant differences in COVID-19 infection rates, severity, duration, and vaccine side effects between these study groups and general population controls.

**Results:**

A panel of 7 IMID experts successfully allocated COVAD-1 diagnoses into single organ and systemic categories; this also directed overlap category membership. Although this work is preliminary and highly exploratory, we anticipate that subsequent analysis will reveal disproportionate levels of severe COVID-19 infection outcomes (hospitalization with and without oxygen support) and vaccine side effects (mild and major) among participants with systemic manifestations of IMID, especially those that qualify for the overlap IMID category.

**Conclusions:**

Advocating for direct-to-patient vaccine reporting pathways, this study intends to produce more precise vaccine safety profiles of patients with IMID. It seeks to resolve current gaps in pharmacovigilance and potentially remedy vaccine hesitancy in high-risk groups by doing so. The international nature of COVAD-1 data collection and the nuance of information made available through participant self-report are to the advantage of this protocol. However, the dependence of this study on participant recall, the small sample sizes handled, and the questionable relevance of these data in the contemporary Omicron era are to the detriment of this work.

**International Registered Report Identifier (IRRID):**

DERR1-10.2196/68785

## Introduction

### Background

The COVID-19 pandemic has had a disproportionate impact on the lives of those with immune-mediated inflammatory diseases (IMID), including autoimmunity [1]. By virtue of their immunosuppressed state, these patients were incorporated into shielding programs where possible and were prioritized for novel medical supplies, including COVID-19 antivirals and vaccines [2]. Such measures were necessary given the heightened rates of hospitalizations, respiratory complications, and mortality observed in patients with IMID who contracted COVID-19 [3,4].

Despite this, COVID-19 vaccine uptake was not universal among patients with IMID and the immunosuppressed more broadly [5]. The absence of these patients from vaccine development trials [6], coupled with the lack of observational studies on their specific vaccine tolerance [7], has generated notable hesitancy in this group [8]. This is visible in comparisons of vaccine refusal rates between key risk groups: work by Gaur et al [9] saw patients with autoimmune disease led chronic risk groups with 19% COVID-19 vaccine refusal versus 17.8% and 13.4% seen among patients with chronic lung disease and cancer, respectively. More precise assessments of vaccine risk in the IMID population are urgently needed to reassure these patients about the safety and suitability of these products [10].

One solution is to enhance pharmacovigilance surveillance. Current methods, including the analysis of computerized medical records or data from centralized reporting mechanisms (Yellow Card, Vaccine Adverse Event Reporting System, etc), are primarily clinician facing and select for major adverse events [11]. It is hypothesized that direct-to-patient vaccine side effect surveys may deliver the detailed and differentiable data that high-resolution pharmacovigilance requires.

The first iteration of the COVID-19 Vaccination in Autoimmune Diseases (COVAD) study, COVAD-1 [12], was established to test this hypothesis, initially identifying whether a patient COVID-19 vaccine survey could provide sufficient data to compare safety profiles between patients with a diverse range of IMID—with a special emphasis on autoimmunity—and general population controls. This protocol, however, describes a follow-on study that will enhance this initial analysis, assessing whether the COVAD dataset can also be used to test for internal heterogeneity in the COVID-19 infection and vaccine experience of the IMID population.

### Study Design

To do so, this work will allocate COVAD respondents with IMID into categories aligned with the electronic Delphi Study to Define and Risk-Stratify Immunosuppression (DESTINIES) phenotype [13]—a COVID-19 risk hierarchy produced and ratified over the course of a multistage international clinical consensus-building exercise of 64 global experts in immunology, vaccinology, and infectious disease (the DESTINIES Consortium). IMID data will be categorized as follows: single organ, systemic, and overlap IMID.

As per the DESTINIES evaluations, we are anticipating that, overall, respondents with IMID will have experienced worse COVID-19 infection and vaccine outcomes than the general population controls. However, internal heterogeneity is highly likely. Following the structure of the DESTINIES phenotype, we also hypothesize that patients with single organ IMID will report milder COVID-19 infections and fewer vaccine side effects than their systemic and overlap counterparts. We also expect that the multimorbidity of the overlap category will predispose this group to the worst outcomes of all those evaluated. However, this investigation is preliminary and highly exploratory; these hypotheses should therefore only be considered as evidence generating at this moment in time.

## Methods

### Overview

COVAD-1 study design, recruitment, data collection, and initial results have been described in detail elsewhere [12]. This multinational cross-sectional survey was active in over 90 countries and accumulated support from a collective of 106 rheumatologists, internists, neurologists, and immunologists. Recruitment used a diverse array of social media channels and online patient support groups globally [14].

Between March and December 2021, the COVAD-1 study collected the following information from vaccine surveys presented to an international cohort of patients with IMID and population-based controls: demographic details, IMID type, treatment details, current symptoms, COVID-19 infection history (inclusive of symptoms, duration, and complications), COVID-19 vaccine details, 7-day COVID-19 adverse events of interest, and specific patient-reported outcome measures (health, pain, activity, fatigue, and physical function status). All personally identifiable data were removed, anonymizing this asset for secondary analysis. Informed consent was obtained at the start of the survey, and no financial incentives were offered for completion.

In total, 18,882 patients with IMID or population-based controls responded to the survey [14]. Self-reported by survey respondents, the following IMIDs were represented in this study set: ankylosing spondylitis or psoriatic arthritis, antisynthetase syndrome, Crohn disease or ulcerative colitis (inflammatory bowel disease), dermatomyositis, hemolytic anemia or idiopathic thrombocytopenic purpura, inclusion body myositis, juvenile dermatomyositis, mixed connective tissue disease, multiple sclerosis, myasthenia gravis, necrotizing myositis, overlap myositis with lupus or Sjögren syndrome or systemic sclerosis or rheumatoid arthritis, pernicious anemia, polymyalgia rheumatica, polymyositis, rheumatoid arthritis, scleroderma, Sjögren syndrome, systemic lupus erythematosus, thyroid disease (hypothyroidism or hyperthyroidism), type 1 diabetes, and vasculitis. Respondents were able to indicate when they had multiple diagnoses.

**Figure 1:**
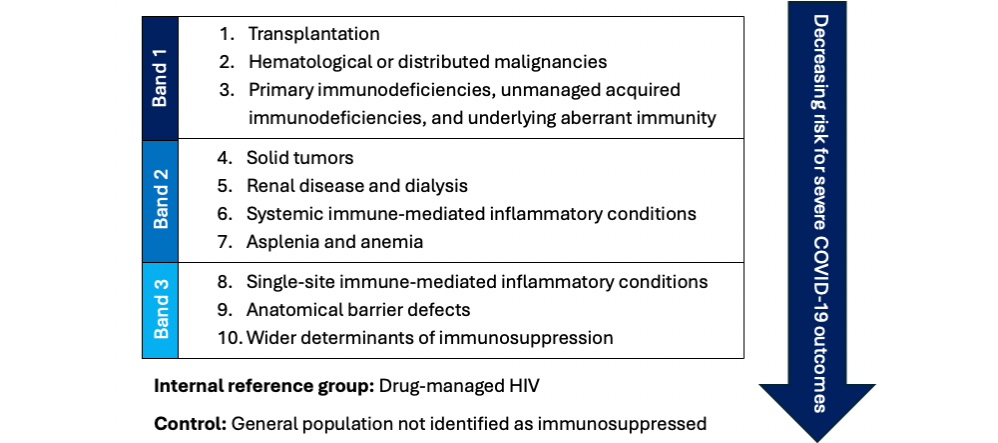
The Delphi Study to Define and Risk-Stratify Immunosuppression (DESTINIES) phenotype.

All survey respondents were older than 18 years, ensuring congruence between this study set and the DESTINIES primary output, the DESTINIES phenotype [13]. Visualized in Figure 1 [13], the DESTINIES phenotype COVID-19 segments the medical diagnoses and procedures that clinical experts agreed confer immunosuppression into 10 risk levels. Here, IMIDs are categorized into single organ and systemic categories, the latter of which is considered at higher risk for severe COVID-19 outcomes. The term single site was updated to single organ to better align with prevailing taxonomies [15].

In our intended investigation, the conditions just cited will be allocated into these categories by relevant experts within the DESTINIES Consortium. Multiple rounds of consensus building will take place if necessary, with 75% agreement as the threshold for consensus in the first round (corresponding with the median consensus value of Diamond et al [16] review of more than 100 successful exercises) and, for any diagnoses that could not be allocated to this consensus threshold, a second round of majority-based allocations will be conducted. Determinations will be made anonymously to reduce risks of bias: this blinding minimizes the influence of panelists’ reputations, personalities, or propensity to defer to group averages [17]. A third IMID category, overlap IMID, will be instituted for survey respondents who self-report both single organ and systemic diseases.

It is anticipated that small sample sizes may preclude detailed comparative analysis, particularly after 3-part IMID segmentation. Incomplete surveys will also be removed from the study set, including those in which respondents do not indicate their IMID status or provide full pharmacovigilance data. This includes respondents who have not received a vaccine dose.

However, statistical power calculations, including minimum sample sizing, were not performed because of the highly exploratory nature of this exercise. The diversity of peer-reviewed outputs that this dataset has produced—including those exclusively focused on subgroup experiences (eg, pregnant or breastfeeding women with autoimmunity) [18]—gives the authors confidence that this work will represent a meaningful contribution to the field.

Furthermore, efforts will be made to preserve usable data where possible, imputing “No response” if questions are skipped. Free-text data, such as that clarifying “Other” selections, will be removed. This includes immune-mediated conditions that fall outside of the 22 diagnoses specified. Google Translate (Alphabet Inc) will be used to ensure eligible diagnoses provided in other languages are not omitted.

To investigate our hypothesis that patients with overlap and systemic IMID experience worse COVID-19 infection and vaccine outcomes than their single organ and general population counterparts, the following analyses will be conducted.

### Descriptive Analysis

Cohort profiling will tabulate and compare IMID categories and the control population by age, gender, ethnicity, country economic status, and self-reported health and physical function levels. Country economic status will be operationalized using World Bank Country classifications [19], whereby “Higher” will refer to countries demarcated as high-income, and “Lower” will refer to those classified as either low, lower-middle, or upper-middle income, accurate as of the 2025 fiscal year. IMID data will be compared with general population controls as an aggregate before being differentiated into systemic, single organ, and overlap categories. COVID-19 vaccine history, including dose count and type, will also be reported. Absolute numbers of COVID-19 infections, hospitalizations (with and without oxygen support), and average case duration and vaccine side effects (major vs mild) will also be tabulated with their supporting summary data. For completeness, efforts will be made to tabulate the frequencies of each specific COVID-19 symptom and vaccine side effect reported and their respective proportions of totals. For vaccine data, results will be presented by each vaccine type (mRNA, adenoviral, inactivated virus, protein subunit, mixed schedules, and unsure) to identify those associated with disproportionately high numbers of side effects or muted outcomes by comparison.

Anticipating small sample sizes, this study will preserve all usable data by not performing age-gender matching. However, as elaborated upon further below, multivariable logistic regression will assess the relative contributions of key demographic and medical variables to the likelihood of a participant reporting hospitalization for COVID-19 or vaccine side effects.

### Simple Logistic Regression for COVID-19 Outcomes

Obtained via simple logistic regression, odds ratios of each outcome of interest will be estimated against the control group reference with their respective 95% CI to assess the influence of study groups on each event. Outcomes of interest include reporting a major vaccine side effect (any, then by each side effect type), reporting a minor vaccine side effect (any, then by each side effect type), COVID-19 infection, COVID-19 hospitalization without oxygen support, and COVID-19 hospitalization with oxygen support.

Anticipating the prospective contributions of vaccine uptake and wider medication and income variables to clinical outcomes, we will perform a variety of subgroup analyses. The above exercise will be repeated but with data segmented by vaccine dose (1 or 2); medication status (received steroid immunosuppressives, did not receive steroid immunosuppressives, received nonsteroid immunosuppressives, or did not receive nonsteroid immunosuppressives); and economic status (higher or lower). For immunosuppressive medication-based subgroup analyses, odds ratios will be calculated using single organ IMID data as the reference category.

### Multivariable Logistic Regression for Assessing the Influence of Covariates

Multivariable logistic regressions will be used to quantify the relative contributions of demographic, health, and vaccination variables on the likelihood of participants self-reporting (1) severe COVID-19 (hospitalization or hospitalization with oxygen support) and (2) vaccine side effects (mild or major) for each population. Survey responses that omit information on vaccine side effects or hospitalization will be withheld from this analysis.

For COVID-19 hospitalization, the following models will be run at the whole cohort level and then separately for IMID (aggregated and categorized) and general population data.

- Model 1 (demographics): age, gender, ethnicity, and country economic status
- Model 2 (demographics+prior health): age, gender, ethnicity, country economic status, health status, and medication status
- Model 3 (demographics+prior health+vaccination): age, gender, ethnicity, country economic status, health status, medication status, and vaccine dose

For COVID-19 vaccine side effects, the following models will be run at the whole cohort level and then separately for IMID (aggregated and categorized) and general population data.

- Model 1 (demographics): age, gender, ethnicity, and country economic status
- Model 2 (demographics+prior health): age, gender, ethnicity, country economic status, health status, and medication status
- Model 3 (demographics+prior health+vaccination): age, gender, ethnicity, country economic status, health status, medication status, vaccine dose, prevaccine symptoms, and medication suspensions

Categorical variables (eg, ethnicity, gender, and self-reported ability to complete daily tasks) will have each of their levels’ influence on COVID-19 vaccine side effect reporting or hospitalization analyzed in turn. This will be assessed by quantifying the impact of changing a category’s reference level (eg, Caucasian [White] for ethnicity data) to test level (eg, Asian) on the likelihood of either event occurring. The reference levels for each categorical variable will be prespecified. The influence of study category on COVID-19 hospitalization and vaccine side effect outcomes will be estimated at the whole cohort and IMID (aggregated) levels, using general population data as the reference in the former instance and single organ IMID data as the reference in the latter instance. All coefficients will be exponentiated and reported as odds ratios with their associated 95% CIs. All findings will be interpreted with the caveats of selection and recall bias that cannot be controlled for in this secondary analysis.

### Ethical Considerations

As a secondary analysis of COVAD-1 data, this protocol adheres to existing COVAD-1 ethical approvals including local institutional ethics committees [20]—Institutional Ethics Committee of Sanjay Gandhi Postgraduate Institute of Medical Sciences (IEC 202-143-IP-EXP-39) [19]—and the Checklist for Reporting Results of Internet E-Surveys [21], which can be accessed in the COVAD protocol [12]. It has the ongoing oversight, endorsement, and participation of the entire COVAD steering group. This protocol pertains to the secondary analysis of anonymized data. Informed consent was obtained before survey completion.

## Results

As a collective, this analysis will identify key demographic differences between IMID categories and general population controls, quantify differences in the COVID-19 infection and vaccine experiences of these groups, and delineate the contributions of numerous demographic and health variables to each group’s likelihood of being hospitalized for COVID-19 or experiencing a side effect after inoculation.

Over the course of the peer review process, this protocol has since been actioned in full; results are now being quality assured by the present authorship. Work began in August 2024, data analysis occurred between November 2024 and July 2025, and journal submission is intended for October 2025. However, this paper will elaborate on results initially presented in this protocol’s preprint edition.

In November 2024, 7 IMID experts within the DESTINIES Consortium were enlisted to categorize COVAD 1 respondents’ diagnoses into their respective study categories (single organ IMID, systemic IMID, or control). Two rounds of consensus building were necessary for this process. However, 7 diagnoses did not meet the initial 75% threshold specified and were entered into the second round of consensus building, where allocations were made by majority vote. The results of these 2 rounds and final categorization are provided in Tables 1 and 2.

Final allocations are provided in Textbox 1. This directed subsequent data cleaning and analysis as specified in the Methods section (active December 2024 to July 2025). Respondents who self-identified as having both single organ and systemic diagnoses according to these categorizations were then allocated into the overlap group.

**Table 1:** Round 1 of consensus building (N=7).

Diagnosis	Single organ, n (%)	Systemic, n (%)	Categorization
Ankylosing spondylitis or psoriatic arthritis	1 (14)	6 (86)	Systemic
Antisynthetase syndrome	0 (0)	7 (100)	Systemic
Crohn disease or ulcerative colitis (inflammatory bowel disease)	2 (29)	5 (71)	Contested
Dermatomyositis	0 (0)	7 (100)	Systemic
Hemolytic anemia or ITP^a^	4 (57)	3 (43)	Contested
Inclusion body myositis	4 (57)	3 (43)	Contested
Juvenile dermatomyositis	0 (0)	7 (100)	Systemic
Mixed connective tissue disease	0 (0)	7 (100)	Systemic
Multiple sclerosis	3 (43)	4 (57)	Contested
Myasthenia gravis	3 (43)	4 (57)	Contested
Necrotizing myositis	3 (43)	4 (57)	Contested
Overlap myositis with lupus or Sjögren syndrome or systemic sclerosis or rheumatoid arthritis	0 (0)	7 (100)	Systemic
Pernicious anemia	4 (57)	3 (43)	Contested
Polymyalgia rheumatica	0 (0)	7 (100)	Systemic
Polymyositis	0 (0)	7 (100)	Systemic
Rheumatoid arthritis	0 (0)	7 (100)	Systemic
Scleroderma	1 (14)	6 (86)	Systemic
Sjögren syndrome	0 (0)	7 (100)	Systemic
Systemic lupus erythematosus	0 (0)	7 (100)	Systemic
Thyroid (hypothyroid or hyperthyroid)	7 (100)	0 (0)	Single organ
Type 1 diabetes	7 (100)	0 (0)	Single organ
Vasculitis	0 (0)	7 (100)	Systemic

^a^ITP: idiopathic thrombocytopenic purpura.

**Table 2:** Round 2 of consensus building (N=7).

Diagnosis	Single organ, n (%)	Systemic, n (%)	Categorization
Crohn disease or ulcerative colitis (inflammatory bowel disease)	2 (29)	5 (71)	Systemic
Inclusion body myositis	4 (57)	3 (43)	Single organ
Multiple sclerosis	5 (71)	2 (29)	Single organ
Hemolytic anemia or ITP^a^	7 (100)	0 (0)	Single organ
Myasthenia gravis	6 (88)	1 (14)	Systemic
Necrotizing myositis	2 (29)	5 (71)	Systemic
Pernicious anemia	6 (86)	1 (14)	Single organ

^a^ITP: idiopathic thrombocytopenic purpura.

**Final single organ and systemic immune-mediated inflammatory disease allocations.**

**Single organ immune-mediated inflammatory disease**

- Type 1 diabetes
- Thyroid (hypothyroid or hyperthyroid)
- Myasthenia gravis
- Multiple sclerosis
- Pernicious anemia
- Hemolytic anemia or idiopathic thrombocytopenic purpura
- Inclusion body myositis

**Systemic immune-mediated inflammatory disease**

- Scleroderma
- Rheumatoid arthritis
- Sjögren syndrome
- Systemic lupus erythematosus
- Overlap myositis with lupus, Sjögren syndrome, systemic sclerosis, or rheumatoid arthritis
- Polymyalgia rheumatica
- Ankylosing spondylitis or psoriatic arthritis
- Vasculitis
- Mixed connective tissue disease
- Antisynthetase syndrome
- Juvenile dermatomyositis
- Dermatomyositis
- Polymyositis
- Crohn disease or ulcerative colitis (inflammatory bowel disease)
- Necrotizing myositis

## Discussion

### Anticipated Findings

Irrespective of whether our data are consistent with the hypotheses specified, an analysis such as this provides valuable insights for clinicians, policymakers, and pharmaceutical firms. For example, any indications of elevated vaccine side effects among specific IMID types, even historical ones, warrant close examination. Alternatively, indications that the COVID-19 vaccine was universally well tolerated among participants with IMID should be made known to patients concerned with taking up this offering going forward. Such evidence would be especially pressing in a context of repeat vaccination, where the risks of experiencing adverse events can accumulate [22].

This direct-to-patient reporting pathway holds immense promise for pharmacovigilance going forward. The COVAD study group has already used this dataset to interrogate multiple longstanding questions in the vaccine benefit-risk remit for patients with IMIDs [23]. It is unique in amplifying the patient voice that is currently missing from pharmacovigilance; by enhancing the granularity of this data, as we intend, this patient survey offers intelligence that could not be procured through routine surveillance alone.

### Limitations

We acknowledge the following limitations in this protocol. First, the COVID-19 vaccine landscape has changed considerably because the COVAD-1 data were collected. At the time of writing, vaccine uptake in certain immunosuppressed categories has since exceeded 10 doses, for example [24]. In comparison, 2 doses are the maximum within this COVAD-1 dataset. This discrepancy could call the relevance of our findings into question; however, their prospective value for enhancing pharmacovigilance and pandemic preparedness efforts counters this assertion.

Second, these data were not collected in real time with vaccination; an accurate picture of 7-day side effects therefore relies on respondents’ recall. To prevent false recall bias, prospective data would have been preferable.

Third, despite the international nature of this work, the sample size is still relatively small—especially once incomplete surveys and the unvaccinated are excluded from the study set and the IMID population is segmented. The imbalanced allocations of IMID diagnoses between single organ and systemic IMID categories are also of concern.

Furthermore, the authors acknowledge that this protocol does little to control for various forms of bias beyond the anonymization of the consensus exercises specified. For example, panelists may have privileged the risk profiling of the patients they are most familiar with, allocating them into the higher risk systemic IMID category—at least by the assessment of the DESTINIES Study [13]. In addition, there was no ability to control for the selection bias inherent to primarily recruiting participants from medical settings; those sick enough to require regular medical supervision may not be representative of the average patient in that population—especially when considered from a perspective of developing economies lens, where medical access is scarcer and can select for the more affluent [25].

Finally, this protocol does not offer any means of controlling for the potential influence of changing COVID-19 variants on infection outcomes. Surveys were completed between March and December 2021, coinciding with the emergence of the Omicron lineage that has since remained dominant [26]. Similarly, any changes in vaccine practices that occurred during this data collection period—such as local preferences for homologous or mixed scheduling [27]—cannot be controlled for. In either case, without survey completion or vaccine administration date data, we are unable to segment the analysis to assess the influence of these changes. We acknowledge that this is detrimental to the rigor of this work.

### Conclusions

At present, patients with IMID are not provided with vaccine safety data that are relevant to their diagnosis. This opacity has been seen to create vaccine hesitancy despite the well-established vulnerability of these patients. This investigation seeks to enhance COVID-19 pharmacovigilance for the IMID population. If successful, this work will corroborate the value of direct-to-patient reporting pathways for vaccine benefit-risk surveillance in complex groups.

## Abbreviations

COVAD: COVID-19 Vaccination in Autoimmune Diseases
DESTINIES: Delphi Study to Define and Risk-Stratify Immunosuppression
IMID: immune-mediated inflammatory disease

## Data Availability

COVID-19 Vaccination in Autoimmune Diseases (COVAD) datasets are not available for public access. Those interested in conducting their own secondary investigations must apply to do so. The COVAD steering group handles these applications on a case-by-case basis.
